# Statistics of Correlations and Fluctuations in a Stochastic Model of Wealth Exchange

**DOI:** 10.3390/e20030166

**Published:** 2018-03-05

**Authors:** Maria Letizia Bertotti, Amit K. Chattopadhyay, Giovanni Modanese

**Affiliations:** 1 Free University of Bozen-Bolzano, Faculty of Science and Technology, I-39100 Bolzano, Italy; 2 Aston University, Mathematics, System Analytics Research Group, Birmingham B4 7ET, UK

**Keywords:** discretized kinetic theory, wealth exchange models, Langevin stochastic equations, multiplicative noise, Ornstein–Uhlenbeck noise

## Abstract

In our recently proposed stochastic version of discretized kinetic theory, the exchange of wealth in a society is modelled through a large system of Langevin equations. The deterministic part of the equations is based on non-linear transition probabilities between income classes. The noise terms can be additive, multiplicative or mixed, both with white or Ornstein–Uhlenbeck spectrum. The most important measured correlations are those between Gini inequality index *G* and social mobility *M*, between total income and *G*, and between *M* and total income. We describe numerical results concerning these correlations and a quantity which gives average stochastic deviations from the equilibrium solutions in dependence on the noise amplitude.

## 1. Introduction

Wealth exchange models [1,2] are used in the context of economic theory and econophysics [3,4] to describe in a simplified way the individual economic interactions occurring in a society. In particular, they allow to predict emerging collective features like the income distribution, the Gini index or the Pareto exponent. Most of these models have equilibrium solutions, but it is also well known that economic systems are never exactly at equilibrium. Hence, fluctuations should also be taken into account in the models. This is in some sense done in economic agent-based models [5,6] which are essentially computer simulations of economic systems based on a population sample. By their very nature they include statistical fluctuations which depend on the size of the sample. In this paper, we shall focus instead on models based on discretized kinetic theory [7,8]. These models are expressed in mathematical form through large systems of non-linear ordinary differential equations which describe transitions of individuals of a society between income classes. The transitions are the consequence of economic interactions which occur with certain probabilities defined by the model and depend on several parameters. Interaction terms can be of degree 2 in the class population densities (direct interactions) or of degree 3 (indirect interactions, used to model redistribution processes). These kinetic models are well established, and have also been tested on networks and for the description of taxation, welfare, tax evasion and tax audits (see e.g., [9,10]).

We have described in [11,12] the complex mathematical procedure needed to introduce stochastic noise into the system and leading to a consistent set of kinetic Langevin equations. The main difficulty in building up this procedure lies in the presence of dynamical constraints which, differently from cases typically treated in the literature, refer to several equations. The stochastic variations of the populations must respect, both in the additive and multiplicative case, the condition that the total population is conserved. In analogy with statistical mechanics, we speak of a “canonical” system (with non-conserved total income), when the total income μ is free to fluctuate and speak of a “micro-canonical” system when the total income is fixed; this requires a second constraint on the stochastic variations. It is also possible to consider a “mixed noise”, such that at each step of the time evolution of the system the stochastic variation is a linear combination with random coefficients of an additive and a multiplicative variation.

In this paper, we present extensive statistical results, almost all relative to a stochastic model with multiplicative noise. They concern quantities which are of major interest for real world economies and whose values today constitute a widespread object of concern [13,14]. Most significant among these quantities is certainly the correlation between economic inequality and social mobility, respectively measured in our model by a parameter *G* which expresses the Gini index and by a suitably defined indicator *M*. The model displays for this correlation, in correspondence with a range of values of the Gini index compatible with those of industrialized countries, negative values (Section 2). This is in agreement with empirical data [15,16]. In Section 3 we investigate the dependence of the correlation $R_{GM}$ on *G*. In Section 4 we briefly discuss the case of Ornstein–Uhlenbeck noise. We also give an estimate of the average deviations of the population from equilibrium as a function $\delta_{av}$ of the noise amplitude Γ (Section 5). Finally, Section 6 contains some conclusions and comments on possible future extensions of the work.

## 2. Simulations for a Fixed Value of the Initial Total Income and Gini Index

### 2.1. Langevin Equation. Deterministic Solutions

**Figure 1:**
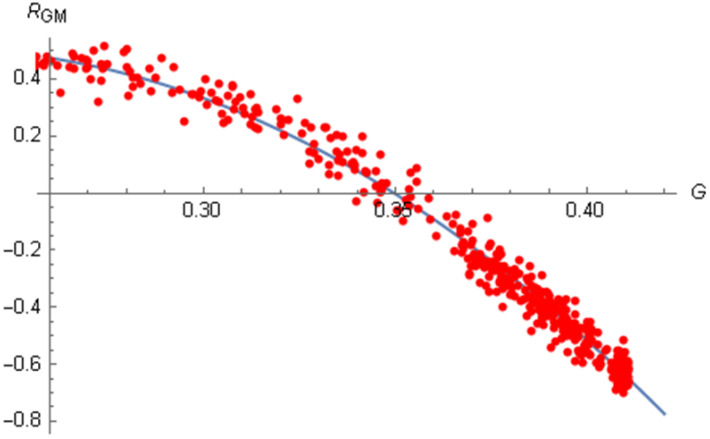
Dependence of the correlation $R_{GM}$ on the Gini index with mixed noise in a range which extends far below the usual values of *G*. Each dot represents the average of 80 realizations with 2500 steps. There are 480 dots in total.

We consider a population of individuals divided into a finite number *n* of classes, each one characterized by its average income $r_{j}$ with $0<r_{1}\leq r_{2}\leq \ldots \leq \;r_{n}$. Let $x_{j}\left(t\right)$ for 1≤j≤n denote the fraction at time *t* of individuals in the *j*-th class. In previous work, see e.g., [8], assuming different economic behaviors of individuals belonging to different classes, two of us constructed a model for the time evolution of $x\left(t\right)=(x_{1}\left(t\right),\ldots ,x_{n}\left(t\right))$ in correspondence to a whole of economic exchanges. The model (actually describing also taxation and redistribution processes) was formulated as a system of ordinary differential equations, in fact “deterministic” in the $x_{j}$ variables. In contrast, the discretized Langevin equations we are dealing with here take the form

(1) $$dx_{i}=\left(\sum_{h,k=1}^{n}C_{hk}^{i}x_{h}x_{k}-x_{i}\sum_{k=1}^{n}x_{k}\right)dt+\Gamma \eta_{i}\sqrt{dt},$$

where $dx_{i}$ represents the variation, in the time interval dt, of the population $x_{i}$ of the income class *i*. The total population is normalized to 1, $\sum_{j=1\ldots n}x_{j}\left(t\right)=1$ for all t≥0. We emphasize here that this normalization, valid at t=0, holds true for all t≥0 as a consequence of the specific choice of the elements entering into the Equation (1). We take the class incomes $r_{i}$ linearly growing in *i* (see example below), even though other choices are also possible. The deterministic variation of $dx_{i}$ (the part proportional to dt) can be easily recognized by setting Γ=0. It contains the coefficients $C_{hk}^{i}$ which define the model by fixing the inter-class transition probabilities and account for the above mentioned behavioral heterogeneity. More precisely, $C_{hk}^{i}$ is the probability that an individual of income class *h* will belong to class *i* after an encounter with an individual of class *k*. These coefficients are required to satisfy the identity $\sum_{i=1}^{n}C_{hk}^{i}=1$ for any h,k∈{1,…,n} and are taken in this paper as in [8]. Note that the transition probability fluxes are proportional to the products $x_{h}x_{k}$ of the class population densities because pairwise monetary exchanges are here considered. The noise vector with components $\eta_{i}$ also must respect the constraints mentioned above. These are implemented through suitable linear transformations applied, at each step in the time evolution, to a vector of stochastic variables with standard Gaussian distributions, see e.g., [12]. The results reported in this paper are all obtained with multiplicative noise, except the plot in Figure 1, which is obtained with mixed noise.

We take as initial condition an equilibrium configuration of the deterministic system with a certain total income $\mu =\sum_{i=1}^{n}r_{i}x_{i}$. Such an equilibrium can be obtained with high accuracy through a Runge–Kutta integration of the deterministic equations over a very long interval (typically $10^{4}$ or $10^{5}$ steps). We recall here that in the deterministic case the equilibrium configuration does not depend on the initial conditions, but only on the total income.

We recall next, before proceeding, the definitions of the quantities which are investigated in the paper.

The *Gini index*, commonly used as a measure of inequality of wealth or income, can range from 0 (complete equality) to 1 (maximal inequality). It can be calculated based on the Lorenz curve, which plots on the axis of ordinates the cumulative percentage of the total income of a population earned by the bottom percentage of individuals, represented on the axis of abscissas. In comparison with it, the 45 degree line represents perfect equality of incomes. The Gini index is defined as the ratio of the area between the Lorenz curve and the 45 degree line and the total area under this line. In our discrete approach, we calculated the area under the Lorenz curve as a sum of trapezia. In the deterministic case, when total income conservation holds true, we calculate the Gini index *G* at equilibrium. In the stochastic case, when total income changes in time, there are no equilibrium solutions and we rather get a time-series for the Gini index.

The *social mobility coefficient* *M* we use here is essentially a weighted average, over the classes, of the probability for an individual to be promoted to the upper class in the unit time. It is computed using an expression first introduced in [17], which can be found e.g., in [11,12] and which we do not report here because it would require a longer definition of symbols entering in the $C_{hk}^{i}$.

Empirical evidence shows a clear correlation between these two quantities. Namely it is found that mobility reduces when inequality rises, thus implying a negative correlation between *G* and *M* [15,16,18]. This correlation, nicknamed the “Great Gatsby Curve” [19], is important since it means that the increase of inequality (as presently observed in several countries) tends to be a self-reinforcing phenomenon, unless it is complemented by suitable social policies. It should also be stressed that this correlation holds for societies at near equilibrium, while it may be different in phases of strong economic growth [20].

Consider now for example a system with 10 classes (n=10), class incomes $r_{i}=10i$, and the coefficients $C_{hk}^{i}$ as in [8]. In order to set the income equal to 30 we can initially put all the population into class 3, i.e., set $x_{3}=1$ and all the other $x_{i}$ equal to zero. To set the income equal to 29, one can assign $x_{2}=0.1$, $x_{3}=0.9$ and all the rest zero, and so on.

**Figure 2:**
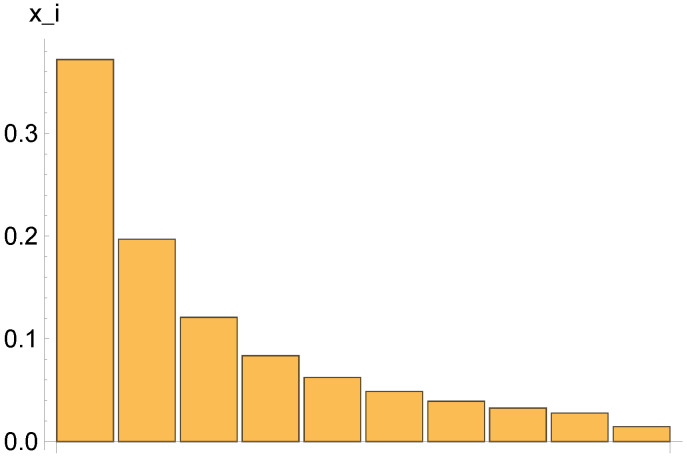
Histogram of a typical deterministic equilibrium configuration, with 10 income classes (see details in the text). The bars represent the populations $x_{1},\ldots ,x_{10}$.

The asymptotic equilibrium configuration with μ=30 is

$$\left\{x_{i}\right\}=\left\{0.372,0.197,0.121,0.0836,0.0623,0.0487,0.0393,0.0326,0.0277,0.0145\right\}$$

(see histogram in Figure 2) and has Gini index $G_{eq}=0.410$ and mobility coefficient $M=5.58\times 10^{-4}$.

**Figure 3:**
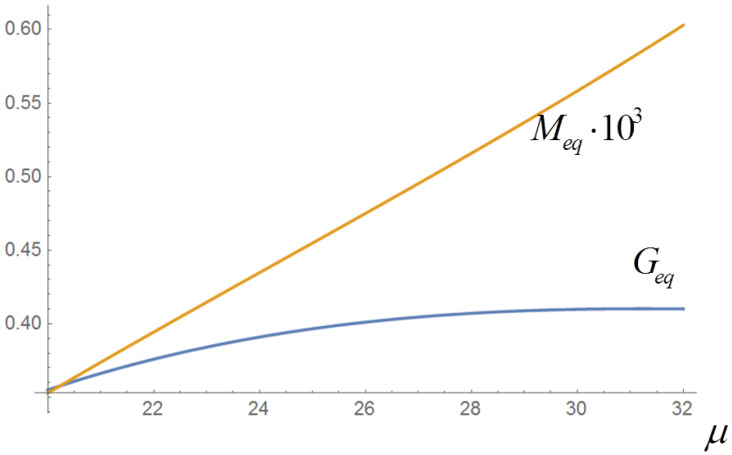
Behavior of the Gini index *G* and the social mobility *M* as functions of the total income μ for the deterministic model, at equilibrium. The asymptotic equilibrium of the deterministic equations does not depend on the detailed initial conditions $x_{i}\left(0\right)$ (class populations), but only on the total income $\mu =\mu \left(0\right)=\sum_{i}r_{i}x_{i}\left(0\right)$ (μ is conserved in the deterministic evolution). This plot is obtained by taking several values of μ(0) as explained in Section 2.1, letting the system evolve deterministically and computing *G* and *M* in the equilibrium state. The relation between *G* and μ allows to determine a range of values of μ which corresponds to a range of realistic values of *G*.

Since the equilibrium configuration depends only on the total income, a biunivocal relation between $G_{eq}$ and μ is defined, which in a reasonable range of $G_{eq}$ is almost linear; one has for instance, in the interval $0.35\leq G_{eq}\leq 0.41$, $G_{eq}=-0.1594+0.03712\mu -0.0006\mu^{2}$ (see Figure 3; the relation between $M_{eq}$ and μ is also shown, although it is not of immediate interest for this work).

### 2.2. Stochastic Time-Series

**Figure 4:**
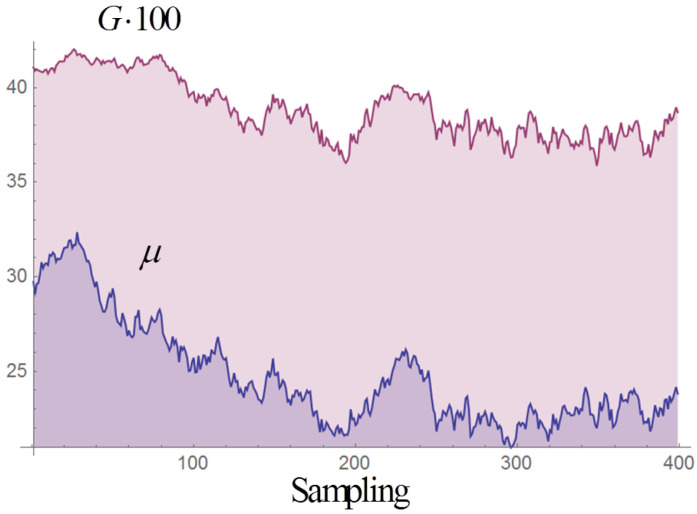
Example of time-series of *G* and μ in the canonical case, with multiplicative white noise of amplitude Γ=0.01. The time steps in the series are 40,000, with a sampling each 100 steps.

**Figure 5:**
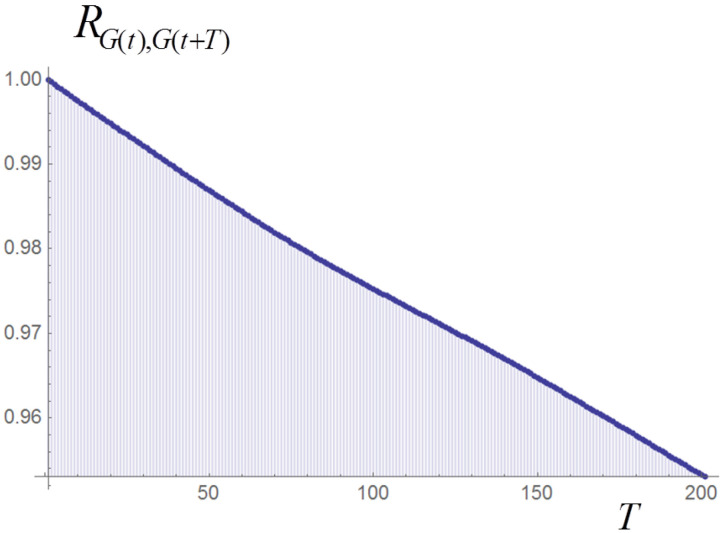
Time auto-correlation of *G* in the time-series of Figure 4. *T* denotes the number of time steps.

In the discretized Langevin Equation (1) we typically set dt=0.1 or dt=1 and let the system evolve in time-series of 5000 steps, repeated for $N_{R}$ realizations; $N_{R}$ varies between 50 and 6000, depending on the scope. (The choices for dt and the number of steps are based on our previous experience with the relaxation time of the deterministic system.) In the following we shall also compare results obtained through time-series of 10,000 integration steps and $N_{R}/2$ realizations, or time-series of 2500 steps and $2N_{R}$ realizations; in principle the results should coincide in the ergodic limit and in fact the averages are very close, but we have found that correlation estimators obtained with the time-series of 5000 or 2500 steps tend to display smaller fluctuations. After each integration step the values of *G* and *M* are computed. Both quantities are non-trivial functions of the populations $x_{i}$. They fluctuate around their equilibrium value according to a Wiener process (see example in Figure 4), as can be checked by evaluating the Hurst exponent of their time-series, which is very close to 0.5. The time auto-correlation function of *G* is remarkably linear (Figure 5). The same is true for the auto-correlation of μ and *M*, though not reported here. (We recall the definition of the Hurst exponent in this context: in a time-series with *N* points, the expectation value of the ratio between the range ρ(N) of the series and its standard deviation σ(N) is proportional to $N^{H}$ as N→∞, where *H* is the Hurst exponent).

**Figure 6:**
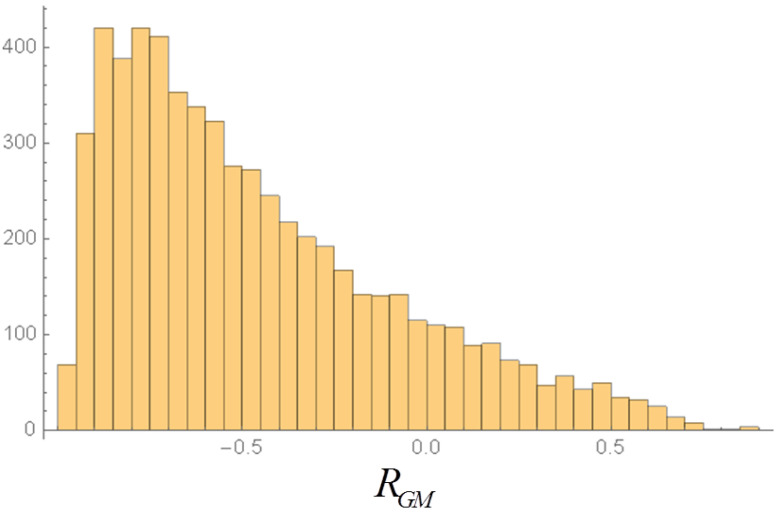
Histogram of the correlation $R_{GM}$ in 6000 realizations of 5000 steps, with multiplicative noise of amplitude Γ=0.001.

**Figure 7:**
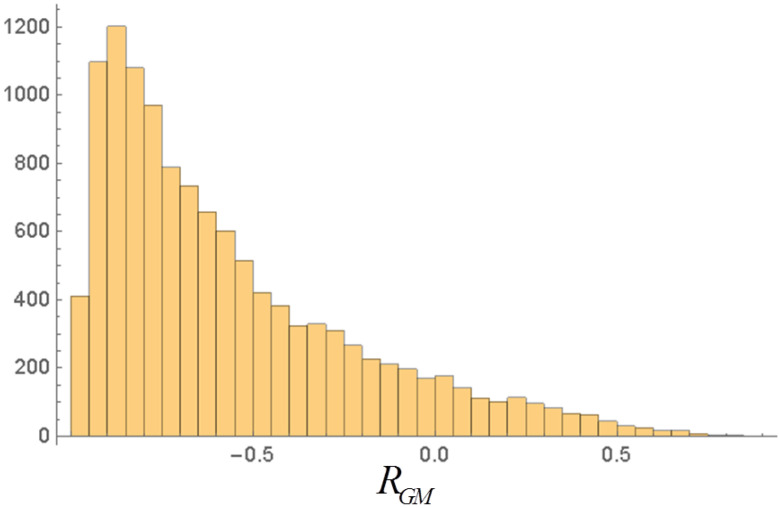
Histogram of $R_{GM}$ in 12,000 realizations of 2500 steps. Compare with Figure 6.

At the end of each time-series we compute the equal time correlation estimators (Pearson coefficients) between *G* and *M*, *G* and μ, *M* and μ. The sign and amplitude of the correlations vary quite strongly in the single realizations. For instance, by performing a very large number of realizations (6000 or 12,000) we find for the results of the *G*-*M* correlation in each realization the histograms in Figure 6 and Figure 7.

These histograms are neither Gaussian nor symmetric. If we regard the values of $R_{GM}$ for each realization as random variables themselves, assuming they are independent we may expect that the averages of $R_{GM}$ over a certain number $N_{R}$ of realizations obey the central limit theorem, and thus have a Gaussian distribution with a standard deviation given by the standard deviation of the single realizations divided by $\sqrt{N_{R}-1}$. In order to check this, we made $N_{S}=200$ series of simulations, each one comprising $N_{R}=50$ realizations of 5000 steps. We obtained in this case $\sigma_{averages}=0.0524$ and $\sigma /\sqrt{N_{S}-1}=0.0531$, which displays a close agreement. The corresponding histogram is reasonably symmetric and Gaussian.

## 3. Dependence of the Correlations on the Total Income and on *G*

In the previous sections we have discussed the statistical properties of the correlations $R_{GM}$, $R_{G\mu }$ and $R_{M\mu }$ in the case of multiplicative noise. The behavior of the correlations was evaluated for a fixed initial value μ of the total income. As reported in [12], however, the value of the correlations has a clear dependence on μ (all other model parameters being fixed). Since there is a biunivocal relation between μ and $G_{eq}$, we can also say that the correlations depend on $G_{eq}$; this choice of variable is actually better, since $G_{eq}$ has a direct economic meaning, while the value of μ depends on the definition of the income classes and on an arbitrary reference unit. Numerical evaluations of the correlations (see [12]) show that in a suitable range of values of μ and $G_{eq}$ the $R_{GM}$ correlation is approximately linear in μ and remains negative, thus confirming the general validity of the empirical “Great Gatsby” rule mentioned in Section 2.1. On the other hand, the correlation $R_{G\mu }$ is approximately linear in μ but changes sign, showing that the question whether in economics “a raising tide lifts all boats” does not have an absolute answer. Finally, the correlation $R_{M\mu }$ is seen to be always very close to 1, confirming the strong linkage between mobility and total income; note that in a physical analogy the total income can be identified with the total energy and thus with the temperature in the canonical case.

Note that for values of *G* much smaller than usual, even the $R_{GM}$ correlation can become positive: see Figure 1, obtained with the mixed noise introduced in [21] and extending to the (unrealistic) value of G=0.25. In order to obtain a meaningful diagram, one needs to generate a large number of distinct initial conditions for the stochastic equations, each one having a different value of μ. For instance, in Figure 1 there are 480 initial conditions, one for each result represented by a red dot. Every result is the average of 80 realizations starting from those initial conditions.

## 4. Langevin Equation with Ornstein–Uhlenbeck Noise and Dependence of the Correlations on Γ and τ

It is straightforward to replace the white noise $\eta_{i}$, used in the Langevin equation until now, with an Ornstein–Uhlenbeck noise $y_{i}$ having memory time τ. To this end, an integration step of the discretized OU stochastic equation is added to each integration step of the Langevin equation. The full integration step, including multiplicative noise normalization, looks as follows:

(2) $$\begin{array}{c} dy_{i}=\left(1-\frac{dt}{\tau }\right)y_{i}+\sqrt{\frac{2dt}{\tau }}\eta_{i} \end{array}$$

(3) $$\begin{array}{c} y_{i}^{1}=x_{i}y_{i} \end{array}$$

(4) $$\begin{array}{c} N=\sum_{k=1}^{n}y_{k}^{1} \end{array}$$

(5) $$\begin{array}{c} y_{i}^{2}=y_{i}^{1}-Nx_{i} \end{array}$$

(6) $$\begin{array}{c} dx_{i}=\left(\sum_{h,k=1}^{n}C_{hk}^{i}x_{h}x_{k}-x_{i}\sum_{k=1}^{n}x_{k}\right)dt+\Gamma y_{i}^{2}\sqrt{dt}. \end{array}$$

**Figure 8:**
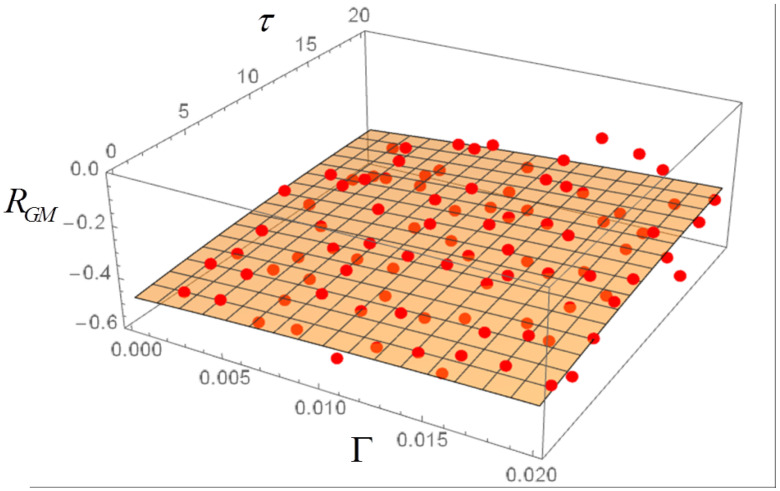
Correlation $R_{GM}$ for Ornstein-Uhlenbeck noise in dependence on noise amplitude Γ and memory time τ. Each dot is the average of 50 realizations with 5000 steps.

**Figure 9:**
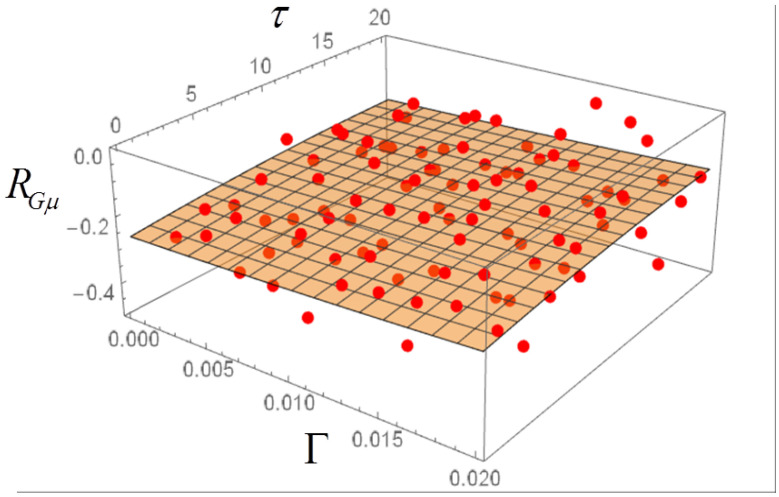
Same as in Figure 8, for the correlation $R_{G\mu }.$

**Figure 10:**
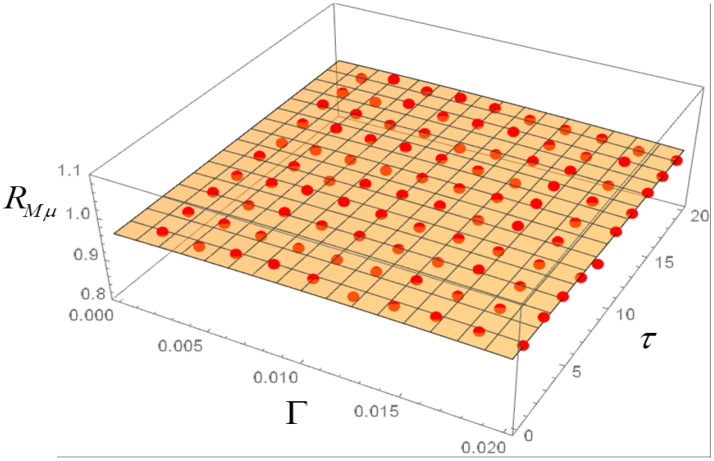
Same as in Figure 8, for the correlation $R_{M\mu }.$

The correlations can now be computed in dependence both on Γ and τ. The plots of Figure 8, Figure 9 and Figure 10 are obtained, where each dot represents the average of 50 realizations. A 3D polynomial fit confirms that the correlations have a very weak dependence on Γ and τ. Note that the largest values of Γ correspond to a very strong noise. The noise amplitude can be related to economic data by considering that for Γ=0.001 the corresponding fluctuations of the total income μ in the stochastic realizations are of the order of 0.1%, thus quite realistic for a society at near equilibrium. The model is robust with respect to an increase in the noise amplitude. For instance, a tenfold increase of Γ leads to a proportional increase in the fluctuations of μ, while the values of the correlations $R_{GM}$, $R_{G\mu }$ and $R_{M\mu }$ are substantially unchanged. For Γ up to 0.032, also with OU noise with memory time τ=32, the fluctuations of μ can be of the order of 50% or more.

## 5. Fluctuations of the Populations in Dependence from Γ

Looking at the equilibrium populations, denoted by $x_{i,eq}$, for certain model parameters and for a certain μ, it is interesting to measure the average amplitude of the stochastic fluctuations $x_{i}-x_{i,eq}$ over several realizations in dependence on the noise amplitude Γ. A suitable measure appears to be the following:

(7) $$\begin{array}{c} \delta_{av}=\langle \sqrt{\sum_{i=1}^{n}\frac{{\left(x_{i}-x_{i,eq}\right)}^{2}}{x_{i,eq}^{2}}}\rangle . \end{array}$$

**Figure 11:**
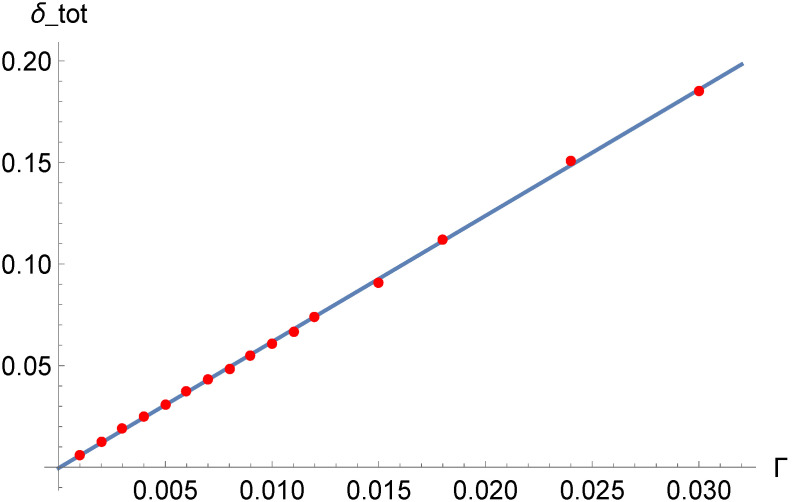
Average deviation $\delta_{av}$ from deterministic equilibrium in dependence on the noise amplitude Γ, for multiplicative white noise. Each dot is the average of 500 realizations with 5000 steps.

As can be seen in Figure 11, the dependence of $\delta_{av}$ on Γ is to a good approximation linear at least up to Γ=0.03, provided the averages are made on a large number of realizations.

## 6. Conclusions

In this work we have investigated the properties of a system of nonlinear Langevin stochastic equations which describe the evolution in time of the income distribution of an idealized society. The individuals of this society interact through money exchanges which are in part deterministic (in the sense that they have fixed transition probabilities) and in part random, being caused by a noise source of the additive, multiplicative or “mixed” kind.

The noise frequency spectrum can be further characterized as white or colored (Ohrnstein–Uhlenbeck noise).

By analysing the results of a large number of numerical simulations we have determined the statistical distribution of the correlations $R_{GM}$ (Gini inequality index—social mobility), $R_{G\mu }$ (Gini—total income), $R_{M\mu }$ (mobility—total income), in correspondence of given average values of the total income. This distribution turns out to be asymmetrical, with a typically negative mean value for $R_{GM}$. On the other hand, the mean value of $R_{M\mu }$ is always positive and close to 1, and that of $R_{G\mu }$ has variable sign, depending on the other parameters of the system.

The dependence of the correlations on the total income μ can be translated into a dependence on *G*, by taking advantage of the deterministic relation between *G* and μ at equilibrium. We have computed the $R_{GM}$ correlation also in a range of inequality values *G* which extends far below the usual range of pre-redistribution *G* values typical of industrialized countries, namely G≥0.35. In this extended range the $R_{GM}$ correlation becomes negative; this means that when inequality is very low, an inequality increase correlates with an increase in social mobility.

In the case of a colored noise with amplitude Γ and memory time τ, the $R_{GM}$ correlation can be computed and plotted as a function of those two parameters, in order to highlight possible transitions between the two regimes $R_{GM}>0$ and $R_{GM}<0$ in the phase plane Γ−τ. No such transitions appear to be present, however. The $R_{G\mu }$ and $R_{M\mu }$ correlations are also slightly affected by variations of Γ and τ.

Finally, the average deviation of the system from the deterministic equilibrium turns out to be a linear function of the noise amplitude, with high accuracy, in a wide range of values of the noise amplitude. This shows that the system is stable with respect to the noise and does not exhibit any tendency to run away from equilibrium, also in the presence of strong noise.

In a conceivable extension of the model, the total population may be allowed to vary. This would remove one of the algebraic constraints and simplify the stochastic version. Immigration and emigration phenomena, or demographic changes on a short or long term could in this way be taken into account. It should be noticed, however, that the deterministic part of the present model changes considerably in the case of a non-constant population, because some general properties of the differential equations cease to be true. We will address these issues in future work.
